# Improved DRUS 4H-SiC MESFET with High Power Added Efficiency

**DOI:** 10.3390/mi11010035

**Published:** 2019-12-27

**Authors:** Hujun Jia, Yuan Liang, Tao Li, Yibo Tong, Shunwei Zhu, Xingyu Wang, Tonghui Zeng, Yintang Yang

**Affiliations:** School of Microelectronics, Xidian University, Xi’an 710071, China; ly_3421@163.com (Y.L.); yibo_tong@126.com (Y.T.); swzhu@stu.xidian.edu.cn (S.Z.); 18844504798@163.com (X.W.); zeng_tonghui@163.com (T.Z.); ytyang@xidian.edu.cn (Y.Y.)

**Keywords:** 4H-SiC MESFET, simulation, power added efficiency (PAE)

## Abstract

A 4H-SiC metal semiconductor field effect transistor (MESFET) with layered doping and undoped space regions (LDUS-MESFET) is proposed and simulated by ADS and ISE-TCAD software in this paper. The structure (LDUS-MESFET) introduced layered doping under the lower gate of the channel, while optimizing the thickness of the undoped region. Compared with the double-recessed 4H-SiC MESFET with partly undoped space region (DRUS-MESFET), the power added efficiency of the LDUS-MESFET is increased by 85.8%, and the saturation current is increased by 27.4%. Although the breakdown voltage of the device has decreased, the decrease is within an acceptable range. Meanwhile, the LDUS-MESFET has a smaller gate-source capacitance and a large transconductance. Therefore, the LDUS-MESFET can better balance DC and AC characteristics and improve power added efficiency (PAE).

## 1. Introduction

As a third-generation semiconductor, silicon carbide (SiC) has significant advantages in materials and devices, with high critical electrical field, high thermal conductivity, and high electron saturation velocity [1,2,3]. SiC based metal-semiconductor field-effect transistors (MESFETs) have become the key components for high-power, high-efficiency and high-frequency microwave applications because of their excellent properties. They offer wider bandwidth operation and lower system size than Si and GaAs based on MESFET technologies [4,5,6,7]. Based on those excellent performance, 4H-SiC MESFETs have broad application prospects in aerospace, satellite communications, and active phased array radars. Previously, the main research on SiC-MESFET was to balance the DC and AC characteristics and improve the output power density. With the idea of energy saving and emission reduction, high efficiency and low energy consumption have become the direction of device design. Thus, some improved structures have been reported for improving the power added efficiency. Such as a novel 4H-SiC MESFET with multi-recessed p-buffer layer for high energy-efficiency applications [8], an improved UU-MESFET with high power added efficiency [9], multi-recessed 4H–SiC metal semiconductor field effect transistor (MRD-MESFET) with high power added efficiency [10], an improved 4H-SiC MESFET with a partially low doped channel [11]. However, the trade-off between PAE and DC AC parameters is still a troublesome problem.

The DRUS-MESFET [12] is proposed to increase the breakdown voltage and improve the output power density of the device based on the double-recessed MESFET (DR-MESFET) [13]. But the saturation current and PAE of the device is low. Considering the saturation current and the power added efficiency, an improved double-recessed 4H-SiC MESFET with partly undoped space region (LDUS-MESFET) is proposed and simulated. The new structure introduced layered doping under the lower gate and optimized the thickness of the undoped region. Using software ISE-TCAD and ADS to simulate the LDUS-MESFET, the DC and AC parameter values and power added efficiency of the device are obtained. By comparing with the DRUS-MESFET, it can be seen that the LDUS-MESFET is more in line with “green high efficiency, energy saving and emission reduction” thoughts.

## 2. Device Structure and Simulation Method and Fabrication Feasibility

### 2.1. Device Structure

**Figure 1:**
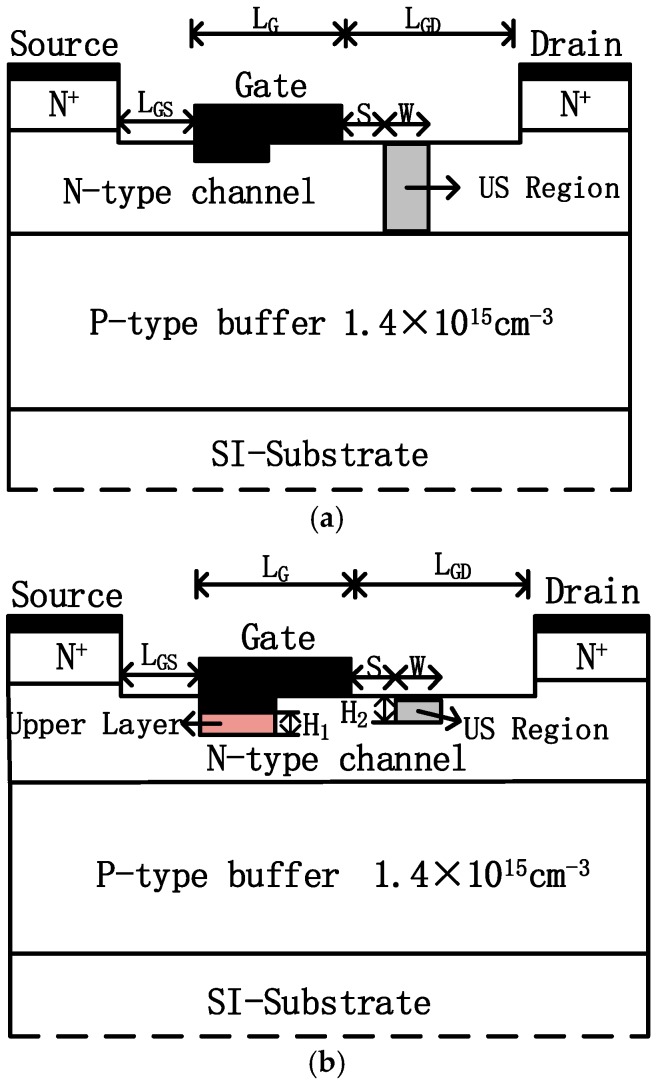
Schematic cross sections of the (**a**) double-recessed 4H-SiC MESFET with partly undoped space region (DRUS-MESFET), (**b**) 4H-SiC metal semiconductor field effect transistor with layered doping and undoped space regions (LDUS-MESFET).

Schematic cross-section of the DRUS-MESFET and the LDUS-MESFET are shown in Figure 1a,b, respectively. They both have a semi-insulating substrate, a p-type buffer layer, an n-type channel layer, and two highly doped n-type cap layers. The semi-insulating substrate is modeled as a compensation-doped (vanadium) semiconductor with a high concentration of deep level impurities [13]. Among them, the role of the p buffer layer is to reduce the influence of substrate defects on the active layer and improve noise performance and device gain [14]. Both of these structures have upper and lower gates, which control a thinner and a thicker part of the channel, respectively. The difference between the two structures is that the LDUS-MESFET has a layered doping under the lower gate, wherein the upper layer region has a doping thickness of *H*_1_ = 0.05 μm and a concentration of 6 × 10^17^ cm^−3^ (to have a meaningful comparison, the lower layer region doping concentration of the LDUS-MESFET based on the DRUS-MESFET is also set at 3 × 10^17^ cm^−3^), and the thickness of the undoped space region (US) is optimized for high efficiency. The optimization result is *H*_2_ = 0.05 μm. The other parameters of the two structures are shown in Table 1.

### 2.2. Simulation Method

**Figure 2:**
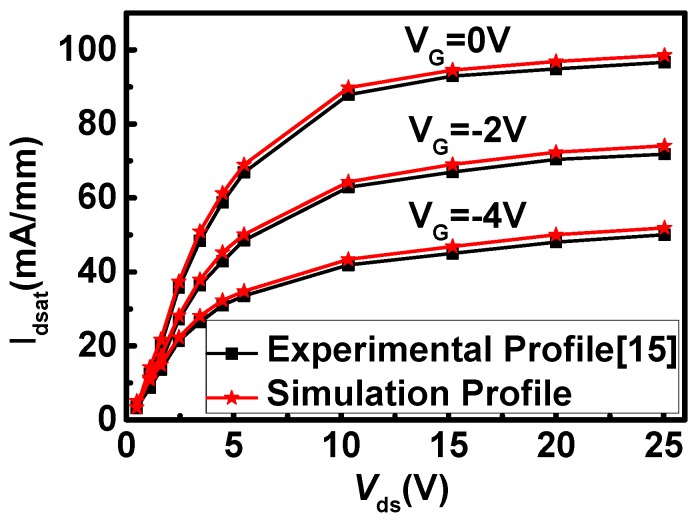
Comparison of experimental data and simulation data on output current.

Two dimensional numerical device characteristics are realized with ISE-TCAD. The simulator is calibrated with experimental data in the micrometer regime [15], and the agreement between experimental data and simulation results is obtained as shown in Figure 2. In order to accurately simulate the electrical characteristics of the 4H-SiC MESFET device structure, the main physical models used are mobility (Enormal, Doping Dep, High Field saturation (GradQuasiFermi)), recombination (Auger, SRH (DopingDep)), incomplete ionization, effective intrinsic density (Band Gap Narrowing (OldSlotboom)). The DC and AC parameters of the device are obtained by ISE-TCAD simulation, and these parameters are input into the ADS software to simulate the power added efficiency of the device. In the ADS simulation, the model used is EE-FET3, an empirical analytical nonlinear model used to fit the electrical properties of MESFETs. And the working bias conditions were set as follows: *V*_gs_ was −3.5 V, *V*_ds_ was 28 V, RF was 1.2 GHz and input power Pavs was 24 dBm.

### 2.3. Fabrication Feasibility

The LDUS-MESFET can be fabricated using the same procedures as reported in [16]. It is worth noting that the doping concentration of 6 × 10^17^ cm^−3^ region under the lower gate can be formed by ion implantation and activation process. Through high temperature and multi-energy ion implantation with phosphorous, and activation of the implanted ions can be achieved by inductively heating at a desired time and temperature in an Ar atmosphere [17]. The formation of undoped space region is formed by high energy ion implantation of deep level vanadium and high temperature annealing (the resistivity of the US region is 2 × 10^6^–7.6 × 10^6^ Ω·cm).

The recessed gate area of the transistors can be fabricated as reported in [16] as follows: First, a thermal oxide layer is deposited on top of the channel, the thermal oxide layer is etched through the position of the recessed area and continues to be etched into the interior of the channel to form a recessed area having a thickness of 0.05 μm. Second, Nickel with a work function of 5.1 eV was deposited on the recessed area to form a Schottky contact.

## 3. Results and Discussion

### 3.1. The Effect of the Doping Concentration (N_d_) and Thickness(H_1_) of the Upper Layer Region on the Device Parameters

**Figure 3:**
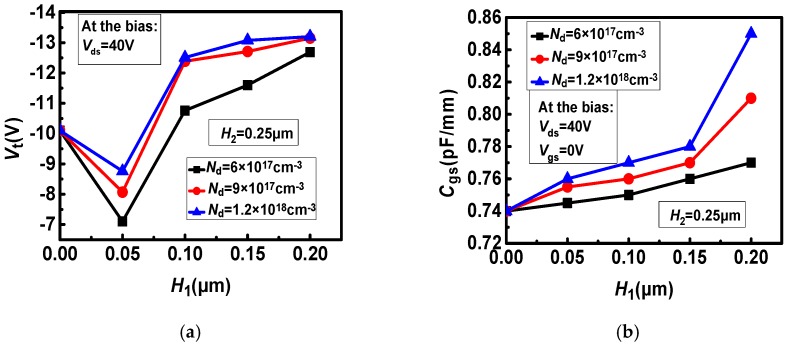

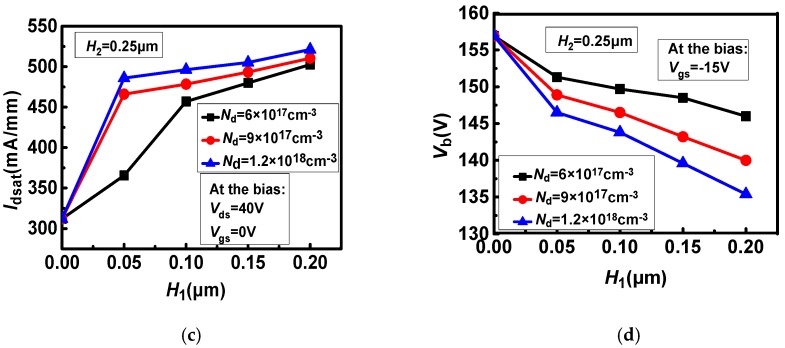
The effect of *H*_1_ and *N*_d_ on the device parameters: (**a**) *V*_t_–*N*_d_ and *H*_1_, (**b**) *C*_gs_–*N*_d_ and *H*_1_, (**c**) *I*_dsat_–*N*_d_ and *H*_1_, (**d**) *V*_b_–*N*_d_ and *H*_1_.

The effect of the thickness and doping concentration of the upper layer region on DC and AC parameters are shown in Figure 3. It can be seen from the Figure 3a that as the doping concentration increases, the absolute value of the threshold voltage increases significantly. When *H*_1_ is less than 0.05 μm, the absolute value of Threshold voltage (*V*_t_) is gradually decreasing with the increase of *H*_1_. Because there is a longitudinal concentration gradient in the channel. The concentration gradient produces a longitudinal electric field that weakens the pinch-off voltage, which results in a decrease in the absolute value of the threshold voltage. When *H*_1_ is greater than 0.05 μm, the absolute value of *V*_t_ is increasing with the increase of *H*_1_. Because as the doping thickness increases, the number of electrons in the channel increases, causing the threshold voltage to drift negatively. At *H*_1_ = 0.05 μm, the absolute value of the threshold voltage has a minimum value. In Figure 3b. As the doping concentration increases, Gate-source capacitance (*C*_gs_) also increases. At the same time, *C*_gs_ shows an upward trend with the increase of *H*_1_. In Figure 3c,d, as the doping concentration increases, the saturation current (*I*_dsat_) increases significantly, and the breakdown voltage decreases. When *H*_1_ rises from 0 to 0.05 μm, the saturation current increases rapidly. When *H*_1_ increases from 0.05 μm, the rising trend of saturation current becomes slow.

### 3.2. The Effect of the Doping Concentration (N_d_) and Thickness of the Upper Layer Region (H_1_) on PAE

**Figure 4:**
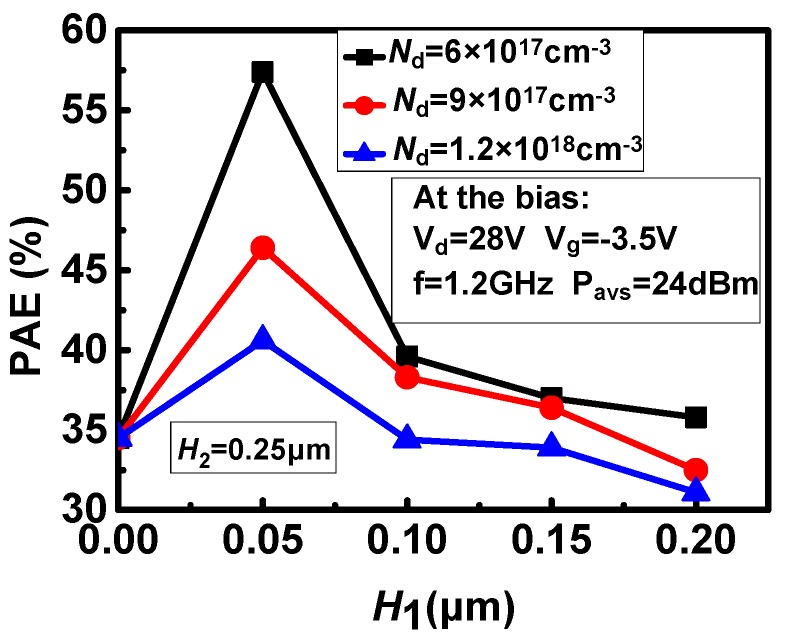
The effects of *N*_d_ and *H*_1_ on the PAE.

The influences of the doping concentration and thickness of upper layer region on PAE is shown in Figure 4. It can be seen from the figure that as the doping concentration of the upper layer region increases, the PAE significantly decreases. When the thickness of the upper layer region is less than 0.05 μm, the PAE increases with the increase of *H*_1_. When *H*_1_ is greater than 0.05 μm, the PAE decreases with the increase of *H*_1_. When the thickness of the upper layer region is 0.05 μm, the PAE reaches a maximum value.

### 3.3. Optimization of the Undoped Region Thickness (H_2_)

**Figure 5:**
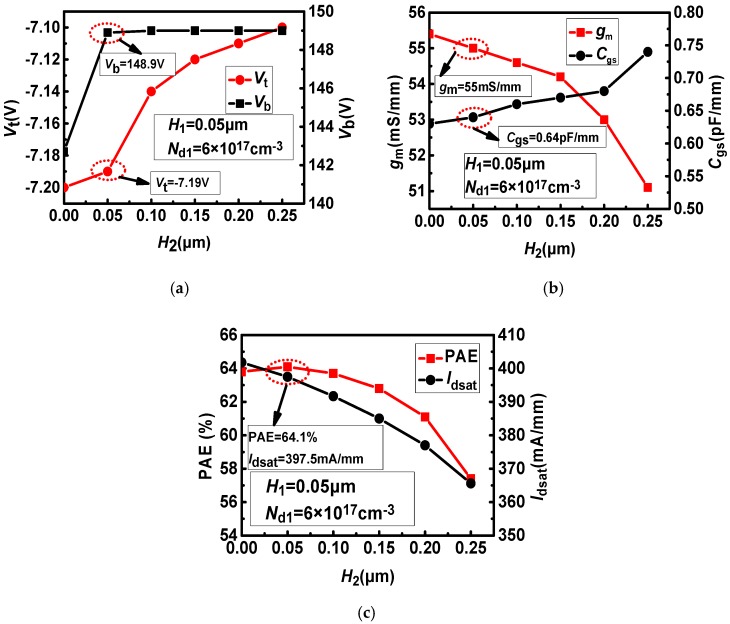
The effect of *H*_2_ on the device parameters: (**a**) *V*_t_, *V*_b_–*H*_2_, (**b**) *g*_m_, *C*_gs_–*H*_2_, (**c**) PAE, *I*_dsat_–*H*_2_.

Based on the thickness and doping concentration of the upper layer region determined above, the thickness of the undoped region is optimized to obtain better power added efficiency and DC AC parameters. The effect of the thickness of the undoped region on the DC, AC parameters and PAE of the device is shown in Figure 5. In Figure 5a. We can see that as *H*_2_ increases, the absolute value of the threshold voltage gradually decreases. At the same time, when *H*_2_ is less than 0.05 μm, the breakdown voltage increases as *H*_2_ increases, when *H*_2_ is greater than 0.05 μm, the breakdown voltage shows a basically constant trend with the increase of *H*_2_. In Figure 5b. As *H*_2_ increases, the transconductance (*g*_m_) shows a decreasing trend. When *H*_2_ is less than 0.15 μm, the transconductance decline trend is more gradual. When *H*_2_ is greater than 0.15 μm, the transconductance decreases sharply as *H*_2_ increases. When *H*_2_ increases, *C*_gs_ shows an upward trend. In Figure 5c. As *H*_2_ increases, the saturation current shows a linear decline. When *H*_2_ is less than 0.05 μm, the PAE increases as *H*_2_ increases, and when *H*_2_ is greater than 0.05 μm, the PAE decreases as *H*_2_ increases. The PAE reaches the maximum at *H*_2_ = 0.05 μm.

### 3.4. Mechanism to Improve Device Parameters

Through the analysis of Figure 4 and Figure 5, it can be acquired that when *H*_1_ = 0.05 μm, *H*_2_ = 0.05 μm, *N*_d_ = 6 × 10^17^ cm^−3^, the power added efficiency reaches the maximum value of 64.1%. It can be seen from Equation (1) that PAE is an efficiency that considers DC and AC parameters [18]. The parameters *V*_t_, *g*_m_ and *C*_gs_ simulated above have an impact on power added efficiency. The larger the absolute value of the threshold voltage of the device, the more difficult it is that the gate voltage is pinched off, which results in higher DC power consumption and smaller PAE. As the maximum transconductance of the device increases, the power-added efficiency of the device tends to decrease linearly. This is because when the device transconductance increases, it means that the ability of the gate to control the current is increased, and the channel resistance is increased, resulting in an increase in the DC power consumption of the device, and the power added efficiency of the device is reduced. The larger the *C*_gs_, the greater the energy lost when charging and discharging the capacitor, resulting in a larger *P*_dc_, which in turn reduces the PAE. The LDUS-MESFET has a smaller threshold voltage and capacitance than the DRUS-MESFET. Although the transconductance is improved compared with the DRUS-MESFET, it can be seen from the simulation that the threshold voltage *V*_t_ and *C*_gs_ have a greater influence on the PAE than the transconductance. So the PAE of the LDUS-MESFET is 85.8% higher than the DRUS-MESFET.

(1) $$\mathrm{PAE}=\frac{P_{\mathrm{out}}-P_{\mathrm{in}}}{P_{\mathrm{dc}}}$$

where *P*_out_ is output power, *P*_in_ is input power and *P*_dc_ is DC power.

It can be seen from Table 2 that the saturation current is increased by 27.4% compared with the DRUS-MESFET. It can be obtained from Equation (2) that the saturation current is proportional to the amount of charge in the channel and the effective thickness of the channel [14]. Heavy doping under the lower gate and reducing the thickness of the undoped region in the channel increase the saturation current. The breakdown occurs at the edge of the gate near the drain side, and the thickness of the undoped region has a certain influence on the breakdown voltage. So the LDUS-MESFET has a lower breakdown voltage than the DRUS-MESFET. But the range of reduction is acceptable. The transconductance reflects the gate voltage’s ability to control the channel current of the device. The partly high doping under the lower gate makes the transconductance of the LDUS-MESFET larger than the DRUS-MESFET. The threshold voltage refers to the gate-source voltage when the channel is pinched off. Since the LDUS-MESFET has a longitudinal concentration gradient in the channel, the concentration gradient produces a longitudinal electric field that weakens the pinch-off voltage, which results in a decrease in the absolute value of the threshold voltage. So the LDUS-MESFET has a smaller threshold voltage than the DRUS-MESFET. Compared with the DRUS-MESFET, the LDUS-MESFET has a reduced thickness of the undoped region near the drain side of the gate. So that the drain side depletion layer can be expanded and the source side expansion is reduced, this makes *C*_gs_ decrease.

(2) $$I_{\mathrm{dsat}}=Q\left(x\right)v\left(x\right)=Zb\left(x\right)qn\left(x\right)v\left(x\right)$$

where *Z* is the channel width, *b*(*x*) is the effective depth of the channel, q is the electron charge, *n*(*x*) is the electron density, and *v*(*x*) is the electron velocity.

## 4. Conclusions

An improved DRUS 4H-SiC MESFET with layered doping under lower gate is proposed and simulated in this paper to increase the PAE of the device. On the basis of the DRUS-MESFET, in order to maximize the power added efficiency, an upper layer region of 0.05 μm thick is introduced under the lower gate, and the thickness of the partly undoped region of the DRUS-MESFET was optimized. The structure achieves a PAE of 64.1%, which is 85.8% larger than the DRUS-MESFET. In addition, the LDUS-MESFET has a certain improve in saturation current, threshold voltage, transconductance, and gate-source capacitance. Although the breakdown voltage is reduced, the drop value is within the acceptable range. Overall, the LDUS-MESFET has wider application in the radio frequency direction.

## Figures and Tables

**Table 1:** Common parameters of the two structures

Parameters	Values
P-Buffer Concentration	1.4 × 10^15^ cm^−3^
N-Channel Concentration	3 × 10^17^ cm^−3^
N-Cap layers Concentration	2 × 10^19^ cm^−3^
Upper layer Concentration	6 × 10^17^ cm^−3^
N-Cap layers Thickness	0.2 μm
N-Channel Thickness	0.25 μm
P-Buffer Thickness	0.5 μm
Recess gate Thickness	0.05 μm
Recess gate Width	0.35 μm
*W*	0.3 μm
*S*	0.2 μm
*L*_gs_	0.5 μm
*L*_gd_	1.0 μm
*L*_s_	0.5 μm
*L*_d_	0.5 μm
*L*_g_	0.7 μm
*H*_1_	0.05 μm
*H*_2_	0.05 μm

**Table 2:** Comparison of performance parameters of the two structures.

Parameters	DRUS-MESFET	LDUS-MESFET
*I*_dsat_ (mA/mm)	312	397.5
*V*_b_ (V)	156.9	148.9
*g*_m_ (mS/mm)	43.8	55
*V*_t_ (V)	−10.1	−7.19
*C*_gs_ (pF/mm)	0.74	0.64
PAE (%)	34.5	64.1
